# Corticospinal tract insult alters GABAergic circuitry in the mammalian spinal cord

**DOI:** 10.3389/fncir.2013.00150

**Published:** 2013-09-25

**Authors:** Jeffrey B. Russ, Tatyana Verina, John D. Comer, Anne M. Comi, Julia A. Kaltschmidt

**Affiliations:** ^1^Weill Cornell/Rockefeller University/Sloan-Kettering Tri-Institutional MD-PhD ProgramNew York, NY, USA; ^2^Neuroscience Program, Weill Cornell Medical CollegeNew York, NY, USA; ^3^Developmental Biology Program, Sloan-Kettering InstituteNew York, NY, USA; ^4^Neurology and Developmental Medicine, Hugo Moser Kennedy Krieger Research InstituteBaltimore, MD, USA; ^5^Neurology, Johns Hopkins School of MedicineBaltimore, MD, USA; ^6^Pediatrics, Johns Hopkins School of MedicineBaltimore, MD, USA; ^7^Cell and Developmental Biology Program, Weill Cornell Medical CollegeNew York, NY, USA

**Keywords:** perinatal stroke, corticospinal tract, GABA interneuron, spinal cord circuitry, presynaptic inhibition, GAD65

## Abstract

During perinatal development, corticospinal tract (CST) projections into the spinal cord help refine spinal circuitry. Although the normal developmental processes that are controlled by the arrival of corticospinal input are becoming clear, little is known about how perinatal cortical damage impacts specific aspects of spinal circuit development, particularly the inhibitory microcircuitry that regulates spinal reflex circuits. In this study, we sought to determine how ischemic cortical damage impacts the synaptic attributes of a well-characterized population of inhibitory, GABAergic interneurons, called GABApre neurons, which modulates the efficiency of proprioceptive sensory terminals in the sensorimotor reflex circuit. We found that putative GABApre interneurons receive CST input and, using an established mouse model of perinatal stroke, that cortical ischemic injury results in a reduction of CST density within the intermediate region of the spinal cord, where these interneurons reside. Importantly, CST alterations were restricted to the side contralateral to the injury. Within the synaptic terminals of the GABApre interneurons, we observed a dramatic upregulation of the 65-isoform of the GABA synthetic enzyme glutamic acid decarboxylase (GAD65). In accordance with the CST density reduction, GAD65 was elevated on the side of the spinal cord contralateral to cortical injury. This effect was not seen for other GABApre synaptic markers or in animals that received sham surgery. Our data reveal a novel effect of perinatal stroke that involves severe deficits in the architecture of a descending spinal pathway, which in turn appear to promote molecular alterations in a specific spinal GABAergic circuit.

## INTRODUCTION

Corticospinal tract (CST) innervation takes place during a critical period of spinal cord maturation and is thought to contribute to a reorganization of spinal circuitry, including the proprioceptive sensorimotor reflex circuit, thereby refining locomotor control (Clowry, 2007). Innervation of the embryonic spinal cord by higher brain centers is one of the final phases of spinal cord development, occurring from late prenatal stages into the perinatal period (Clowry, 2007). Since communication between the cortex and spinal cord is established during this period, the descent of the CST and its incorporation into sensorimotor circuitry is particularly vulnerable to ischemic insults such as perinatal stroke.

Perinatal stroke occurs in 1 of every 4000 term births (Lynch and Nelson, 2001) and may lead to diverse neurologic morbidities, including a range of motor deficits (Ganesan et al., 2000), suggesting altered output from the spinal cord as a potential component of the underlying pathophysiology. These alterations in spinal circuit function may arise from damage to the CST, as the presence and severity of motor impairments have been associated with degeneration and atrophy of the CST (Domi et al., 2009; van der Aa et al., 2011). Attempts at characterizing changes in the spinal cord from loss of supraspinal input after spinal cord transection or stroke have focused on global changes in neurotransmitter systems or functional changes in sensorimotor transmission (Tillakaratne et al., 2000; Hubli et al., 2012). However, it remains unknown how perinatal CST disruption affects specific spinal circuits at the synaptic level, particularly with regard to the inhibitory interneuronal populations that modulate sensorimotor output.

In this study, we investigated how loss of CST in a mouse model of perinatal stroke (Comi et al., 2004) results in specific changes in a presynaptic inhibitory microcircuit of the spinal cord that modulates the efficacy of sensory terminals in the sensorimotor reflex circuit. In this circuit, position and stretch information is carried into the spinal cord via proprioceptive sensory neurons that synapse onto motor neurons (Mears and Frank, 1997). A population of inhibitory, GABAergic interneurons, called GABApre neurons, form contacts on the sensory afferent terminals to specifically inhibit their signaling, via a mechanism called presynaptic inhibition (Rudomin and Schmidt, 1999; Hughes et al., 2005; Betley et al., 2009). Presynaptic inhibition is thought to silence extraneous sensory input during voluntary movement, refine the spatial and temporal perception of incoming sensory information, and prepare a contracting muscle for its impending load (Meunier and Pierrot-Deseilligny, 1998; Seki et al., 2003; Rudomin, 2009). These crucial functions mediated by presynaptic inhibition of sensory terminals are often disrupted in patients with perinatal cerebral injury (Achache et al., 2010).

We show that CST terminals contact putative GABApre neurons in the spinal cord and that unilateral ischemic damage to the cortex, via carotid artery ligation, results in diminished CST synaptic density in the region of the spinal cord where the GABApre neurons reside. We find that CST damage is associated with an upregulation in GABApre interneurons of their synaptic GABAergic machinery. These results provide insight into how changes in spinal inhibitory microcircuitry might influence motor output and recovery after perinatal stroke.

## RESULTS

### DESCENDING CORTICAL PATHWAYS FORM CONTACTS ON PUTATIVE GABAPRE INTERNEURONS

**FIGURE 1:**
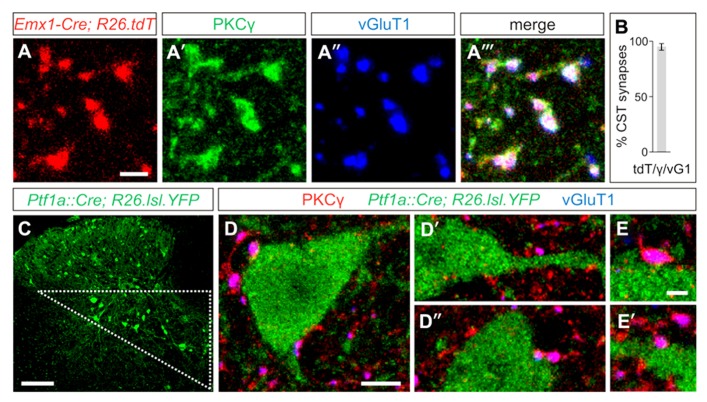
**Putative GABApre interneurons receive corticospinal input.** **(A–A^‴^)** In postnatal day (P)23-26 *Emx1-Cre;ROSA26^CAG-lox-STOP-lox-tdTomato/+^* mice, the tdT reporter **(A)** co-labels with the synaptic terminal markers PKCγ **(A′)** and vGluT1 **(A^″^)**, indicating the specificity of this combination of markers for labeling CST terminals in the spinal cord. **(B)** Quantification of tdT^ON^/vGluT1(vG1)^ON^ terminals that co-label with PKCγ(γ). **(C)** Putative GABApre interneuron cell bodies can be genetically labeled in *Ptf1a^Cre/+^;ROSA26^lox-STOP-lox-EYFP/+^* mice, and reside in the intermediate region of the spinal cord (triangle). **(D–D″)** Numerous examples of CST synapses are observed adjacent to YFP^ON^ cell bodies and nearby dendrites in the intermediate region of P21 *Ptf1a^Cre/+^;ROSA26^lox-STOP-lox-EYFP/+^* spinal cords. **(E,E′)** Higher magnification of CST synapses on YFP^ON^ interneurons. Scale bars: 2 μm in **(A–A‴,E,E′)**, 100 μm in **(C)**, 5 μm in **(D–D″)**.

In order to visualize the synaptic terminals of layer V cortical neurons that descend through the CST into the spinal cord, we examined the selective immunolabeling of CST terminals in the mouse spinal cord. We labeled CST terminals through co-localization of the markers PKCγ, a serine/threonine kinase, and the vesicular glutamate transporter, vGluT1 (Hantman and Jessell, 2010). To quantify the specificity of these markers, we labeled the CST genetically by crossing *Emx1-Cre* mice (Gorski et al., 2002) with a tdTomato (tdT)-reporter line *ROSA26^CAG-lox-STOP-lox-tdTomato^* (Madisen et al., 2010), wherein Cre-mediated recombination of loxP sites drives the expression of the tdT reporter to label descending input from the cortex. Gating our analysis to definitive CST terminals, identified as tdT^ON^/vGluT1^ON^ terminals, we observed a high degree of specificity in the overlap between these two markers and PKCγ (**Figures 1A–A‴**), with 95 ± 3% of tdT^ON^/vGluT1^ON^ synapses also co-expressing PKCγ (*n* > 700 terminals, three mice; **Figure 1B**). No PKCγ/vGluT1 co-labeling was observed independent of a tdT^ON^ terminal, reinforcing the specificity of this combination of markers for visualizing CST terminals in the spinal cord. Thus, co-expression of PKCγ and vGluT1 is sufficient to label CST synapses in the mouse spinal cord.

To determine whether CST synapses form on GABApre interneurons, we made use of the previously identified molecular and anatomical characteristics of this subset of GABAergic interneurons. GABApre interneurons express the transcription factor Ptf1a, and in the ventral spinal cord, *Ptf1a^Cre^*-driven reporters co-label with the GABApre synaptic markers GAD65 and GAD67 (Glasgow et al., 2005; Betley et al., 2009). We previously showed that expression of a *Ptf1a^Cre^*-driven reporter overlaps with a *GAD65-GFP* transgene predominantly in cell bodies located in the intermediate region of the spinal cord, as does co-expression of *Gad2* (GAD65) and *Gad1* (GAD67; Betley et al., 2009). Moreover, GAD65-GFP^ON^ synapses in the ventral horn can be retrogradely traced to cell bodies in the intermediate spinal cord (Hughes et al., 2005). Since these studies suggest that GABApre interneuron cell bodies reside within the intermediate region of the spinal cord, we investigated the presence of CST input onto YFP^ON^ interneurons in the intermediate region of *Ptf1a^Cre/+^;ROSA26^lox-STOP-lox-EYFP/+^* spinal cords (**Figures 1C–E′**). We observed that 79 ± 6% of YFP^ON^ interneurons receive input from PKCγ^ON^/vGluT1^ON^ CST terminals (*n* = 118 cells, three mice). Importantly, this may underestimate CST input onto YFP^ON^ interneurons, as numerous examples of CST synapses on YFP^ON^ dendrites in this area were apparent that could not be traced to a particular YFP^ON^ cell body and were therefore not included in the analysis. Given that a high percentage of YFP^ON^ interneurons in the intermediate region of *Ptf1a^Cre/+^;ROSA26^lox-STOP-lox-EYFP/+^* spinal cords are contacted by CST synapses, this suggests that GABApre interneurons receive descending cortical input.

### CORTICAL INJURY DIMINISHES CST SYNAPTIC DENSITY IN THE INTERMEDIATE REGION OF THE SPINAL CORD

We next aimed to determine how CST innervation density on putative GABApre interneurons would be affected by severe cortical injury. We induced a unilateral injury to the cortex at postnatal day (P)12 via carotid artery ligation, an established model for neonatal ischemic cortical injury (Comi et al., 2004). P12 was chosen as an appropriate murine correlate of the perinatal stage in humans from the perspective of the developing CST. In humans, CST innervation of the spinal cord begins prenatally and continues to develop in the perinatal period (Eyre et al., 2000). However, in mice, this process begins postnatally and does not extensively invade the lumbar gray matter until after P8 (Gianino et al., 1999; Bareyre et al., 2005).

**FIGURE 2:**
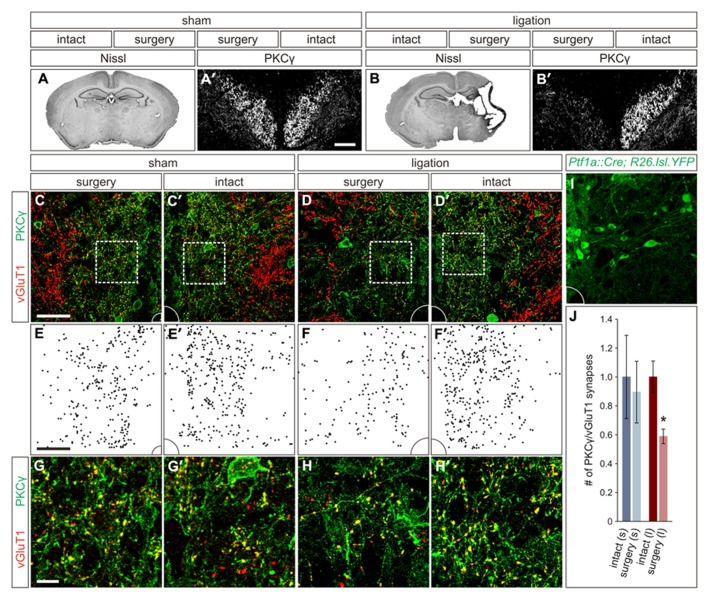
** Carotid artery ligation results in unilateral decreased innervation of the CST to the intermediate region of the spinal cord. (A,A′)** In P21 sham surgery animals, no cortical atrophy is present **(A)** and PKCγ^ON^ CST axons can be visualized coursing bilaterally through the dorsal funiculus **(A′)**. **(B,B′)** In P21 carotid artery ligation animals, the injured hemisphere exhibits severe cortical atrophy **(B)**, and PKCγ staining in the dorsal funiculus is unilaterally lost **(B′)**. **(C,C′)** In sham surgery animals, a comparable density of CST terminals is observed in the intermediate region of the spinal cord on both the surgery **(C)** and intact **(C′)** sides. **(D,D′)** In carotid artery ligation animals, the density of CST terminals is reduced on the surgery side of the spinal cord **(D)** as compared to the intact side **(D′)**. **(E–F′)** Density plots, **(E,E′)** and **(F,F′)**, of CST terminals in the intermediate regions shown in **(C,C′)** and **(D,D′)**, respectively. **(G–H′)** Higher magnification, **(G,G′)** and **(H,H′)**, of the boxed regions shown in **(C,C′)** and **(D,D′)**, respectively. **(I)** Intermediate region of *Ptf1a^Cre/+^;ROSA26^lox-STOP-lox-EYFP/+^* spinal cord, showing numerous YFP^ON^ putative GABApre cell bodies and dendrites, that corresponds to the full panels shown in **(C–D′)**. **(J)** Quantification of CST synaptic density in intact and surgery sides of sham surgery (s) and carotid artery ligation (l) animals. *P ≤ 0.05. Scale bars: 50 μm in **(A′,B′–F′,I)**, 10 μm in **(G–H′)**.

Seizure scores in the ligation-injured group averaged 39 ± 7 and correlated strongly with severity of the brain atrophy (ten mice; Spearman’s rho = 0.796, *p* < 0.01). Quantification of the infarct volume in the injured hemisphere of the brain revealed a mean of 41 ± 5% atrophy compared to the hemisphere contralateral to injury (**Figure 2B**). In contrast, when comparing ipsilateral and contralateral hemispheres in mice that received sham surgery, the mean atrophy was negligible (eight mice; 0.1 ± 1.4% atrophy, *p* < 0.001; **Figure 2A**) and no seizures were noted, similar to what was previously described for this model (Comi et al., 2004).

While the span of injury was large and often affected subcortical structures, including the basal ganglia and hippocampus, the cortex is the only structure within the damaged region that makes direct connections with the spinal circuitry, via the CST. Therefore, we next examined the damage to the CST in these mice, which in sham surgery controls can be visualized coursing bilaterally through the dorsal funiculus of the spinal cord using an immunostain for PKCγ (**Figure 2A′**; Mori et al., 1990). In animals that underwent carotid artery ligation, a unilateral loss of PKCγ staining contralateral to the injured cortical hemisphere was observed (**Figure 2B′**). Diminished staining was not observed in the dorsal funiculus contralateral to the intact hemisphere (**Figure 2B′**). Thus, unilateral ischemic cortical injury leads to a contralateral loss of layer V cortical pyramidal cell axonal projections through the CST.

With such an apparent injury-induced loss of CST axons, we next asked how the innervation density of CST terminals in the relative location of GABApre cell bodies is altered in spinal cords of the carotid artery ligation model. We quantified PKCγ^ON^/vGluT1^ON^ CST terminals in the intermediate spinal cord region of P21 mice that underwent either sham or carotid artery ligation surgery. In the sham surgery animals, no significant difference was noted in the number of CST terminals on the side of the spinal cord contralateral to surgery as compared to the intact side (three mice; *p* = 0.513; **Figures 2C,C′,E,E′,G,G′,J**). However, in animals that underwent carotid artery ligation surgery, a 41 ± 5% reduction in the number of CST synapses was observed in the intermediate region of the spinal cord contralateral to the injured hemisphere as compared to the intact side of the spinal cord (three mice; *p* = 0.05; **Figures 2D,D′,F,F′,H,H′,J**). Examination of the corresponding intermediate regions in the spinal cords of *Ptf1a^Cre/+^;ROSA26^lox-STOP-lox-EYFP/+^* mice showed numerous YFP^ON^ cell bodies and dendrites (**Figure 2I**), supporting the inference that the observed reduction in CST innervation would be particularly significant in the same region where putative GABApre interneurons reside. Thus, ischemic cortical injury results in a lack of descending CST axonal projections as well as a relative decrease in CST terminal density in the area of putative GABApre interneurons, likely affecting their innervation by the CST.

### CST INSULT LEADS TO ALTERATIONS IN GABAPRE SYNAPTIC PROTEIN EXPRESSION

**FIGURE 3:**
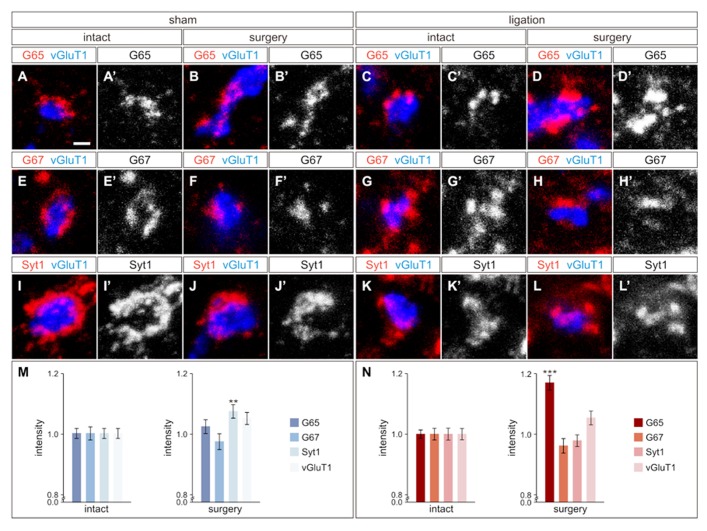
**GABApre terminals upregulate GAD65 in response to cortical injury.** **(A–D′)** GAD65 (G65) localization in GABApre terminals in sham surgery animals [intact side **(A,A′)**, surgery side **(B,B′)**], and in carotid artery ligation animals [intact side **(C,C′)**, surgery side **(D,D′)**]. **(E–H′)** GAD67 (G67) localization in GABApre terminals in sham surgery animals [intact side **(E,E′)**, surgery side **(F,F′)**], and in carotid artery ligation animals [intact side **(G,G′)**, surgery side **(H,H′)**]. **(I–L′)** Syt1 localization in GABApre terminals in sham surgery animals [intact side **(I,I′)**, surgery side **(J,J′)**], and in carotid artery ligation animals [intact side **(K,K′)**, surgery side **(L,L′)**]. **(M,N)** Quantification of G65, G67, Syt1, and vGluT1 fluorescence intensity levels in GABApre terminals of sham surgery animals **(M)** and carotid artery ligation animals **(N)**. **P ≤ 0.01; ***P ≤ 0.001. Scale bar: 1 μm in **(A–L′)**.

As ischemic cortical injury leads to reduced cortical input into the region of the spinal cord where GABApre neurons reside, we next asked if this reduction would in turn affect GABApre neuronal circuitry. GABApre terminals in the ventral horn of the spinal cord can be visualized by immunostaining for pairwise combinations of the synaptic markers GAD65, GAD67, and Synaptotagmin-1 (Syt1) adjacent to a vGluT1^ON^ proprioceptive sensory terminal (Betley et al., 2009). No change in the number of GABApre synapses per sensory terminal was observed between the intact and surgery sides of the spinal cord in ligation or sham animals (*n* > 300 sensory terminals, three mice; *p* = 0.94 for sham animals, *p* = 0.26 for ligation animals). We next examined whether loss of CST input to the intermediate region of the spinal cord resulted in alterations to the fluorescence intensity levels of GABApre synaptic proteins. After carotid artery ligation surgery, GAD65 levels in GABApre terminals were upregulated by 17 ± 2% on the side of the spinal cord contralateral to injury as compared to the intact side (*n* > 300 terminals, five mice; *p* < 0.001; **Figures 3C–D′, N**). No change, however, was noted in the levels of the other GABApre synaptic markers, GAD67 and Syt1, between the intact and injured sides (*n* > 300 terminals, five mice; *p* = 0.21 for GAD67, *p* = 0.43 for Syt1; **Figures 3G–H′, K–L′, N**). In sham control animals, intensity levels for all markers were not significantly different between the two spinal cord hemisections, other than a small difference in Syt1 levels (*n* > 300 terminals, seven mice; *p* = 0.42 for GAD65 and GAD67, *p* < 0.01 for Syt1; **Figures 3A–B′, E–F′, I–J′, M**). Also, as a control neuronal population that does not receive cortical input, the level of vGluT1 intensity in proprioceptive sensory terminals was unchanged between the surgery and intact sides of the spinal cord for both sham and ligation animals (*n* > 300 terminals, five ligation mice; *p* = 0.07; seven sham mice; *p* = 0.06; **Figures 3M,N**). These data indicate that diminished CST input results in an upregulation of GAD65 expression in GABApre terminals, specifically on the side of the spinal cord contralateral to injury. The observed increase in GAD65, but not GAD67, may be part of a mechanism to increase inhibitory potency at the GABApre terminal, as GAD65 produces a readily mobilized pool of synaptic GABA in addition to the general levels typically maintained by GAD67 (Asada et al., 1997; Tian et al., 1999; Engel et al., 2001).

## DISCUSSION

In the developing mammalian spinal cord, the CST plays a crucial role in shaping mature spinal circuitry. In order to begin to understand how developmental disruption of the CST affects specific spinal inhibitory microcircuits, we use a mouse model of perinatal stroke to examine changes in the synaptic properties of a population of presynaptic inhibitory GABAergic interneurons, called GABApre neurons. We observe CST input onto putative GABApre interneurons in the intermediate region of the spinal cord and show that a unilateral carotid artery ligation disrupts the density of CST innervation in the intermediate region of the spinal cord contralateral to injury. With the unilateral loss of CST input, we show in GABApre terminals a corresponding upregulation of the synaptic GABA synthetic enzyme GAD65 on the side of the spinal cord contralateral to the cortical injury. That this upregulation of GAD65 occurs specifically on the surgery side of the spinal cord, and not the intact side, supports the idea that this effect is due to the unilateral disruption of the CST, rather than more general systemic changes.

To establish and maintain precise locomotor control, the development of spinal circuitry and the incorporation of input from higher regions of the nervous system, such as the cortex, are intricately coordinated processes that ultimately must achieve and sustain a delicate balance of inhibition and excitation within sensorimotor circuits. Our observation of CST input onto putative GABApre interneurons in the intermediate region of the spinal cord suggests that the CST can impact the specific inhibitory microcircuitry that controls the proprioceptive sensorimotor reflex circuit. We therefore propose that the upregulation of GAD65 that we observe in GABApre terminals in our mouse model of perinatal stroke is a direct consequence of decreased CST input. This raises the question of why GABApre interneurons would respond to CST loss by upregulating GAD65 at their synaptic terminals. One possibility is that this upregulation reflects an attempt to appropriately readjust the inhibitory balance in the sensorimotor circuit in response to the diminished CST excitation of GABApre interneurons. Synthesizing a higher concentration of GABA at the synapse might help maintain adequate inhibitory strength with each incidence of vesicle release (Engel et al., 2001). It is interesting to note that this increase is unique to GAD65 and is not observed for GAD67, which may reflect functional differences between the two isoforms. For instance, GAD65 is thought to produce the majority of readily releasable synaptic GABA, beyond the baseline levels of GABA more generally produced by GAD67 (Asada et al., 1997; Tian et al., 1999). However, the functional consequences of increased synaptic GABA production in GABApre terminals remain to be determined.

In humans, perinatal cerebral damage, often through stroke, has been found to lead to a variety of neurological impairments with locomotor sequelae including motor deficit, lack of coordination, and even hemiplegic cerebral palsy (Ganesan et al., 2000; Achache et al., 2010), and in many cases the presence of motor impairments can be predicted by the degree of damage to the CST (Domi et al., 2009; van der Aa et al., 2011). While various mouse models of neonatal ischemic brain injury demonstrate functional and motor impairments when subjected to the Rotarod test (Ten et al., 2004), the open field test (Ten et al., 2004), and developmental reflex testing (Ten et al., 2003), other studies of perinatal stroke report no observable motor deficits (Kadam et al., 2009). One possible explanation for this discrepancy is compensation from other descending supraspinal pathways, as rodents exhibit functional compensation from subcorticospinal pathways after CST lesions (Kanagal and Muir, 2009). Moreover, compensatory branching from CST axons on the intact side of the spinal cord could help to retain gross motor function on the surgery side. Previous anterograde tracing studies have demonstrated that perinatal cortical damage in rodents can induce bilateral sprouting from the intact CST into the denervated side of the spinal cord, and it is speculated that this may assist in recovery of motor function (Rouiller et al., 1991; Aisaka et al., 1999). Indeed, compensatory sprouting might explain why we only observe a 41% reduction in CST synaptic density on the injured side of the spinal cord, despite an almost complete loss of CST axonal staining in the dorsal funiculus on the injured side. Future studies may help parse out whether newly formed connections from sprouted CST axons play a role in altering the spinal inhibitory microcircuitry after cortical injury.

The interpretation of results from current mouse models of perinatal stroke when compared with clinical data from human patients relies on both the timing of the initial injury and the age of analysis. In human patients that suffer perinatal cortical damage, few initial movement abnormalities are detectable, and the developmental consequences of the initial injury often do not become apparent until later stages (Ferrari et al., 1990; Leonard et al., 1991; Bouza et al., 1994). It is therefore possible that while motor function is relatively preserved in our mouse model of perinatal stroke, as reported by Kadam et al. (2009), longer-term studies could ultimately reveal aberrant motor behaviors. In this regard, the results of our P21 analysis of the inhibitory microcircuitry may demonstrate a subacute response of GABApre interneurons to CST disruption that is distinct from more chronic changes that might ultimately emerge much later after perinatal stroke. Another variable that may also have consequences for how GABApre interneurons are affected by cortical damage is the age of injury. For example, patients that suffer a stroke as an adult exhibit weakened presynaptic inhibition (Lamy et al., 2009). While our perinatal stroke data support a model of strengthened output from GABApre interneurons, it remains unknown how presynaptic inhibition of proprioceptive sensory neurons is ultimately modified overall. Despite differences in the functional and behavioral outcomes of mice and humans that experience cortical ischemic injury, the observed impact on GABApre circuit development that we describe in mice may provide insight into the specific spinal circuit alterations that occur after perinatal stroke. Future electrophysiological and behavioral studies will be necessary to resolve these data with the clinical data from human patients, as well as to fully understand how the observed changes in spinal cord inhibitory microcircuitry impact motor function and recovery after injury.

## MATERIALS AND METHODS

### MOUSE STRAINS

*Emx1-Cre* (Gorski et al., 2002), *Ptf1a^Cre^* (Kawaguchi et al., 2002), *ROSA26^CAG-lox-STOP-lox-tdTomato^* (Jackson, Ai14; Madisen et al., 2010), and *ROSA26^lox-STOP-lox-EYFP^* (Srinivas et al., 2001) were described previously. Both male and female mice were used in this study; *n* ≥ three mice for all experiments. Experiments have been approved by and conform to the regulatory standards of the Institutional Animal Care and Use Committee of Memorial Sloan-Kettering Cancer Center.

### CAROTID ARTERY LIGATION SURGERY AND TISSUE HARVESTING

P12 CD1 mice received permanent double-unilateral ligation of the right carotid artery under isofluorane anesthesia and the outer skin was re-closed, as described previously (Comi et al., 2004). Sham control animals were treated identically except the carotid was neither dissected nor ligated. After surgery, pups were allowed to recover in an incubator at 36.5°C for 4 h before being returned to the mother. During this time behavioral seizure scoring was performed as previously described (Comi et al., 2004). Ligated mice with high seizure scores were selected for further study. At P21, animals were anesthetized before being transcardially perfused with 4% paraformaldehyde (PFA). The brain was excised from the skull and post-fixed in 4% PFA, cryoprotected in sucrose, and snap frozen. Spinal cords were dissected via ventral laminectomy and processed for immunohistochemistry. Analysis of transverse spinal cord sections was limited to lumbar segments L1–L4.

### IMMUNOHISTOCHEMISTRY

Immunohistochemistry on 12 μm transverse sections of lumbar spinal cords (L1–L4) was performed as preciously described (Betley et al., 2009). The following antibodies were used in this study: rabbit anti-GAD65 (1:8000; Betley et al., 2009), rat anti-GAD65 (1:3000; Betley et al., 2009), rabbit anti-GAD67 (1:10000; Betley et al., 2009), sheep anti-GFP (1:1000; Biogenesis), rabbit anti-PKCγ (1:500; Santa Cruz), mouse anti-Syt1 (1:100; ASV48, Developmental Studies Hybridoma Bank), and guinea pig anti-vGluT1 (1:32000; Betley et al., 2009).

The following secondary antibodies were used for fluorescence intensity analysis: 488-labeled donkey anti-rat (1:1000; Invitrogen), 488-labeled donkey anti-mouse (1:1000; Invitrogen), Cy3-labeled donkey anti-rabbit (1:800; Jackson ImmunoResearch), DyLight 649-labeled donkey anti-guinea pig (1:500; Jackson ImmunoResearch).

### CORTICAL ATROPHY AND SPINAL CORD SYNAPTIC QUANTIFICATION

#### Cortical atrophy quantification

For analysis of cortical atrophy, MicroComputer Imaging Device (MCID; InterFocus Imaging Ltd, Cambridge, UK) was used to measure hemispheric areas of 40 μm, Nissl-stained coronal sections spanning rostral striatum to caudal hippocampus, as previously described (Comi et al., 2004). The hemispheres of each analyzed section were outlined separately, and the areas were calculated based on a pixel threshold value that differentiates between brain and background. Atrophy was calculated for each section as follows: [1 - (area of hemisphere ipsilateral to injury/area of hemisphere contralateral to injury)] × 100 = percentage ipsilateral atrophy. The values from each section were averaged to calculate the hemispheric brain atrophy for each brain.

#### Synaptic quantification

To assess the specificity of the synaptic markers PKCγ and vGluT1 for labeling CST terminals, images were collected using a Leica SP5 confocal microscope, and terminals were quantified using counting software developed in the lab. To quantify PKCγ^ON^/vGluT1^ON^ CST input onto putative GABApre interneurons, CST terminals were gated to YFP^ON^ somata or dendrites in the intermediate region of *Ptf1a^Cre/+^;ROSA26^lox-STOP-lox-EYFP/+^* spinal cords.

To investigate how carotid artery ligation surgery affects the density of CST terminals in the intermediate region of the spinal cord where putative GABApre interneurons reside, the number of PKCγ^ON^/vGluT1^ON^ CST terminals within a defined area of the intermediate region was quantified for sham and carotid artery ligation animals. The absolute number of CST terminals on the surgery side of either sham or ligated spinal cords was then normalized to the mean number of terminals on the intact side of the spinal cords.

#### Synaptic intensity measurements

Synaptic marker intensity measurements were determined using Leica Application Suite Advanced Fluorescence (LASAF) imaging software. For GABApre marker intensity measurements, GABApre terminals were included for analysis if they abutted a vGluT1^ON^ sensory terminal. Sensory terminals for vGluT1 intensity measurements were included for analysis if they were contacted by a GABApre terminal. Three-dimensional optical stacks were collapsed, and terminals were identified and traced in two dimensions, each as an individual region of interest (ROI). Stacks were then un-collapsed for intensity analysis in three dimensions, and the maximum mean intensity value for synaptic terminal markers within each ROI was collected. Intensity data for either sham surgery or carotid artery ligation hemisections were normalized to the mean intensity of control data from intact hemisections.

#### Statistical analysis

Correlation of seizure scores with atrophy quantification was calculated using Spearman’s rho. Differences in CST synaptic density counts in the intermediate region of the spinal cord and differences in the number of GABApre terminals per sensory terminal were calculated using a Wilcoxon rank-sum (Mann–Whitney) test. Differences in immunofluorescence intensity levels were calculated using a Student’s *t*-test. The significance threshold was *p* ≤ 0.05. Results are reported as mean value ± SEM.

## Conflict of Interest Statement

The authors declare that the research was conducted in the absence of any commercial or financial relationships that could be construed as a potential conflict of interest.
